# Color Vision Deficits and Binocular Vision Dysfunction in Parkinson’s Disease

**DOI:** 10.3390/brainsci16020213

**Published:** 2026-02-11

**Authors:** Julia Mleczek, Anim Forjindam, Aasef Shaikh, Fatema Ghasia

**Affiliations:** 1 School of Medicine, Case Western Reserve University, Cleveland, OH 44106, USA; 2 Daroff Dell’Osso Ocular Motility Laboratory, Cleveland VA Medical Center, Cleveland, OH 44106, USA; 3 Louis Stokes Cleveland VA Medical Center, Cleveland, OH 44106, USA; 4 Ocular Motility & Vision Neurosciences Laboratory, Cleveland Clinic, Cleveland, OH 44106, USA

**Keywords:** Parkinson’s disease, color vision, eye movement, vergence, binocular vision

## Abstract

**Background/Objectives:** Visual dysfunction is a common non-motor symptom in Parkinson’s Disease (PD), as evidenced by deficits in color vision (CV) and binocular vision (BV). Computerized CV tests, such as the Cambridge Color Test (CCT), are underutilized in this patient population despite the known limitations of common CV tests. **Methods:** In total, 19 PD and 12 control participants underwent a comprehensive eye exam, including ocular motility testing and the CCT, utilizing thresholds obtained along 12 contrast vectors to fit a discrimination ellipse. Findings were compared across groups, and the association with disease severity was analyzed. **Results:** PD participants showed increases in ellipse area (*p* = 0.012) and short-axis length (*p* = 0.009). PD participants demonstrated convergence insufficiency type exotropia (*p* < 0.001) and impaired stereopsis (*p* = 0.006). No significant correlation with UPDRS scores was seen for either BV or CV. **Conclusions:** PD participants exhibited binocular vision dysfunction with selective changes in color vision. CV changes are more variable in PD, likely due to mixed parvocellular and cortical dysfunction. Convergence insufficiency type exotropia is more common in PD, likely due to combined cortical and subcortical neurodegeneration. Both BV and CV changes occur independently of motor severity, emphasizing the need for routine visual testing regardless of symptom progression.

## 1. Introduction

Parkinson’s Disease (PD) is one of the most common neurodegenerative disorders worldwide, primarily characterized by progressive dopamine (DA) depletion in the basal ganglia, leading to bradykinesia, resting tremor, and rigidity [1]. In addition to these hallmark motor features, there is growing recognition that PD encompasses a broad spectrum of non-motor symptoms. Among these, visual dysfunction has emerged as a significant—and often under-recognized—component of the disease [2].

Visual complaints are common in individuals with PD, with many patients reporting symptoms including blurred or double vision, difficulty judging distances, watery eyes, and even visual hallucinations [3,4]. These disturbances can significantly impair quality of life, with many patients identifying visuospatial difficulties as a major contributor to disability in activities of daily living [5,6]. Clinically, visual dysfunction in PD arises from both sensory and oculomotor abnormalities [7], and importantly, some visual changes have been detected in the prodromal stages of the disease, raising interest in their potential diagnostic value [8,9]. A substantial body of evidence highlights basal ganglia–oculomotor pathway involvement in PD. The basal ganglia form dense reciprocal connections with the superior colliculus, brainstem vergence generators, and frontal and parietal eye fields—networks that support saccadic control, smooth pursuit, fixation, and binocular coordination. Accordingly, oculomotor deficits, particularly those affecting binocular vision, are well documented [10,11]. Convergence insufficiency (CI) is commonly reported in PD [7,12,13,14,15,16,17] and may occur even without overt diplopia [13], and additional binocular deficits, including reduced stereopsis, are frequently noted [18,19]. These findings align with the strong mechanistic link between basal ganglia dysfunction and eye-movement control.

In parallel, sensory visual abnormalities have also been reported in PD, including impaired color discrimination. However, findings across studies are less consistent. For example, although screening Ishihara testing may appear normal in many PD patients [20], more comprehensive measures like the Farnsworth–Munsell 100 Hue Test (FMT) often reveal subtle but meaningful abnormalities [21]. This test asks participants to organize colored tiles in a range of hues to assess their ability to distinguish color and hue changes, with high error rates indicating possible color vision deficiency [21]. Using the FMT as a measure for color discrimination, PD patients exhibit impaired color discrimination [22,23,24,25]; this impairment also correlates with disease severity [23] and duration [24], and has been ameliorated with anti-Parkinsonian medication [26]. However, the need for initiation and control of voluntary motor movements to arrange the color tiles, a process that is impaired in PD, makes this testing strategy less ideal for color vision assessments in this patient population. Studies that show high error rates in the PD population and amelioration with medication administration could be potentially due to changes in motor skills [27] rather than changes in color discrimination. This dilemma has made tests with lower motor demands necessary to fully elucidate how PD affects color vision.

The Cambridge Color Test (CCT) offers a potential solution to this dilemma, providing computerized color vision testing with much lower motor demand. Here, participants are presented with disks of varying luminescence, creating a pseudo-isochromatic Landolt “C” ring. This target is oriented in one of four directions (left, right, up, or down), and the participant is instructed to select the corresponding directional key [28]. Research on color vision deficits in PD using the CCT is limited. One study of 30 PD patients using this technique found heterogeneous patterns of color vision change and independent impairment along the Protan (red, L-cone) and Deutan (green, M-cone) axes in PD [29]. However, another study using this technique found no significant difference in color vision between PD and control groups, though they noted some individual PD patients with significantly different thresholds compared to controls [27]. However, none of the studies on CCT have simultaneously evaluated binocular visual dysfunction.

Thus, the primary objective of the present study was to evaluate sensory and binocular visual function in individuals with PD, using the CCT to assess color vision with reduced motor demand and clinical measures to assess binocular function. We hypothesized that individuals with PD would exhibit impairments in both domains relative to controls, with more pronounced deficits in binocular vision due to the strong and well-characterized involvement of basal ganglia circuits in eye movement and vergence control, compared with the more variable sensory deficits arising from retinal and early visual pathway abnormalities. Finally, we examined whether visual performance was associated with disease severity by relating CCT and binocular visual function outcomes to Unified Parkinson’s Disease Rating Scale (UPDRS) scores.

## 2. Materials and Methods

### 2.1. Participants

In total, 19 participants with Parkinson’s Disease [ages: 52–79] were included. Treatment of these participants included 11 with Deep Brain Stimulation (DBS) and 8 on anti-Parkinsonian medication regimens. PD participants with DBS were evaluated in the DBS-ON state, and PD participants on medication regimens were evaluated in their medication-ON state. A total of 12 age-matched healthy control participants [ages: 56–81] were used in total for group comparisons. Data was collected at the Ocular Motility and Vision Neurosciences Laboratory at the Cole Eye Institute in Cleveland, Ohio.

### 2.2. Experimental Setup

#### 2.2.1. The Cambridge Color Test

**Figure 1:**
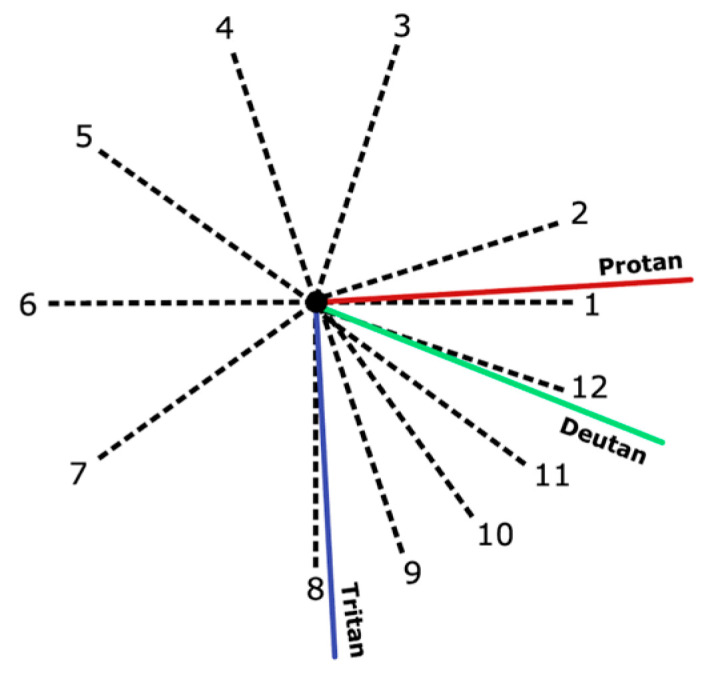
Approximate positions of Protan (red, L-cones), Deutan (green, M-cones), and Tritan (blue, S-cones) color confusion lines on the u′v′ diagram. Numbers indicate the vector number, corresponding to contrast vector locations in a 12-vector test. (Image modified from Metropsis Manual [28]).

In this experiment, color vision was measured using the CCT—Ellipse Test [28]. Color discrimination was tested along 12 discrimination vectors from a central reference point in the u′v′ space (Figure 1). For each discrimination vector, participants are presented with the CCT stimuli in an adaptive staircase mechanism to establish the participants’ maximum sensitivity to chromatic difference. The default test protocol presents the stimuli on screen for a maximum of 3 s; after this presentation window, subsequent stimuli were not presented to the participant until a response was selected. In this study, potential confounding effects of suboptimal motor ability on response speed were mitigated by having the participant verbally report the orientation of the stimuli, with the clinical researcher inputting the participant’s response. Color discrimination vector thresholds (mean and standard deviation) are established as the smallest perceivable color difference for the participant, and are reported in the CIE u′v′ diagram. A larger vector length indicates poorer discrimination ability along that vector. This was repeated across 12 different vectors. The threshold values of these vectors were plotted to produce a MacAdam ellipse (“discrimination ellipse”) in the u′v′ diagram, with the ellipse representing the boundaries of a participant’s color discrimination.

Beyond individual vector length, variables of interest included ellipse long-axis length, ellipse short-axis length, and ellipse area. Here, these values were scaled by 10^3^ for reporting purposes, following previous styles [28]. The angle of the long-axis of the ellipse was also recorded and used in our analysis. When plotted, a larger ellipse area generally indicates weaker color discrimination. The ellipse’s long axis length typically represents the discrimination threshold for the vector that the participant had the most difficulty discriminating relative to the other vectors. Higher axis ratios (normal < 2.0 [28]) indicate selective color discrimination deficits in a specific direction. The angular orientation of the ellipse represents the orientation of the major axis of the ellipse, and can be utilized to understand the type of color vision loss [30]. For example, an axis rotated ~−90 degrees would indicate poor discrimination along the Tritan axis, suggesting blue-yellow color vision deficiency.

#### 2.2.2. Binocular Vision Testing

Binocular vision was measured by clinical assessment. Variables of interest were: strabismic angle of deviation, near point of convergence, and stereopsis ability. The alternating prism cover test at near was used to assess for strabismus, with units reported in prism diopters. Vergence deficits were identified using the Royal Air Force ruler (RAF) to identify the near point of convergence (NPC) [31]. Stereopsis was evaluated using the Titmus Stereo Test [32], utilizing stereoscopic plates to test gross to fine stereoacuity, increasing precision from ~3000 arcsec to <60 arcsec. Log-adjusted values were used in the analysis. Severity of PD was assessed by the MDS—Unified Parkinson’s Disease Rating Scale (MDS—UPDRS) [33].

### 2.3. Data Analysis

Statistical analysis was completed with GraphPad Prism 10 [34] and IBM SPSS Statistics (Version 30.0.0) [35]. Summary statistics (means and standard deviations) were calculated for all demographic characteristics and outcome variables. Group comparisons for color discrimination vectors were conducted (PD: N = 17; controls: N = 11) using repeated measures ANOVA. Welch’s *t*-tests with Bonferroni corrections were used for group comparisons for the remaining CCT variables (ellipse area, as well as long- and short-axis lengths). Chi-squared analysis was used to compare ellipse angle measurements between groups. Independent sample *t*-tests with Bonferroni corrections were completed for binocular vision variables. One tailed test was used as our a priori hypothesis was directional and supported by prior literature. Spearman’s rank correlations were used to assess relationships between CCT results and UPDRS scores, and binocular vision testing results and UPDRS scores.

## 3. Results

### 3.1. Demographic and Clinical Information

Control participants had an average age of 69.58 (±7.40). Of the PD participants, there was an average age of 67.53 (±8.88) with an average UPDRS score of 17.68 (range: 6–43). There was no significant difference in age between PD patients and controls (t = −0.668, two-sided *p* = 0.510, and d = −0.246).

### 3.2. Color Vision

#### 3.2.1. Overall Ellipse Characteristics

**Figure 2:**
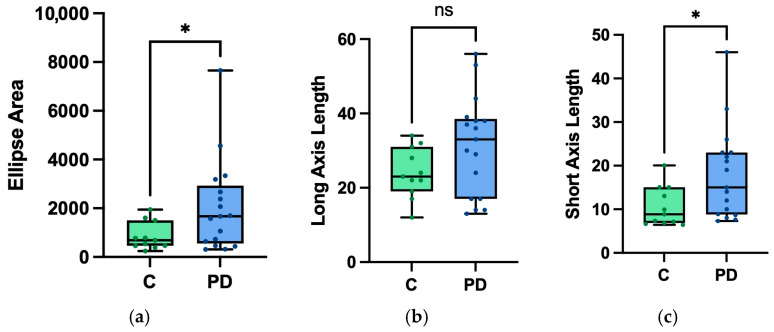
Plots of mean and standard deviation of control participants (“C”) vs. PD participants (“PD”) for the (**a**) ellipse area, (**b**) long-axis length, and (**c**) short-axis length. All values are reported in CIE 1976 u′v′ units, scaled by 10^3^. Statistical significance was established using Welch’s *t*-test with a significance level of *p* < 0.0167. (* = *p* < 0.0167, ns = not significant).

The area of the color discrimination ellipse was increased in PD participants when compared to control participants (t = 1.907, *p* = 0.012, d = 0.780) (Figure 2). The ellipse short-axis length also significantly increased in PD participants compared to controls (t = 2.548, *p* = 0.009, d = 0.847) (Figure 2). There was no significant change in the ellipse’s long-axis length between the two groups (t = 1.907, *p* = 0.034, d = 0.647) (Figure 2).

**Figure 3:**
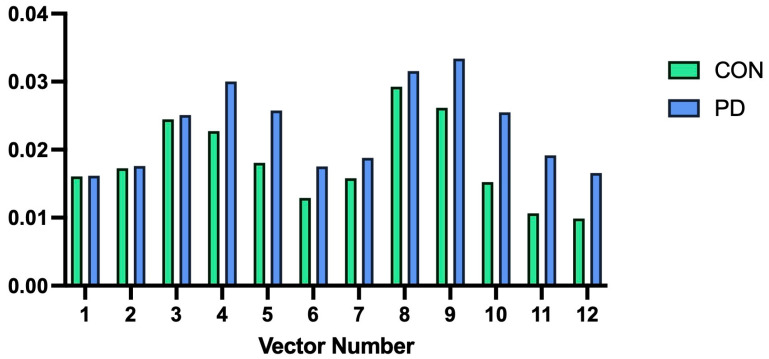
Plot of mean color discrimination vector lengths for control participants (Left bars, “CON”) versus Parkinson’s disease participants (Right bars, “PD”). Length is reported in CIE 1976 u′v′ units.

**Figure 4:**
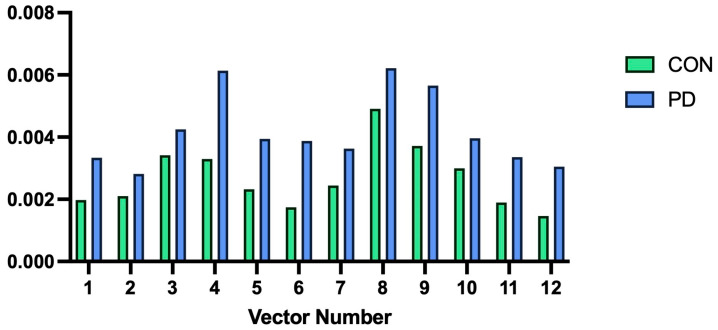
Plot of mean standard deviation of color discrimination vector lengths for control participants (Left bars, “CON”) versus Parkinson’s disease participants (Right bars, “PD”). Length is reported in CIE 1976 u′v′ units.

We also examined differences in mean vector length across 12 color discrimination vectors using repeated-measures ANOVA. There was a near-significant main effect between groups (F = 4.076, *p* = 0.054, η_p_^2^ = 0.136). With a *p*-value approaching significance, we explored within-subject effects with Greenhouse–Geisser corrections, finding no significant effect of group status (PD vs. controls) on vector means (F = 0.946, *p* = 0.450, η_p_^2^ = 0.450) (Figure 3). Repeated-measures ANOVA was repeated for the standard deviation of color discrimination vectors, with the main effect between groups approaching significance (F = 3.007, *p* = 0.095, η_p_^2^ = 0.104). As the *p*-value again approached significance, within-subject effects with Greenhouse–Geisser correction were explored; there was no significant effect of group status on vector standard deviations (F = 0.539, *p* = 0.718, η_p_^2^ = 0.020) (Figure 4).

We also evaluated the ellipse angle across controls and PD participants. The majority of control participants (63%) had an ellipse angle < 90°, whereas the remaining had an ellipse angle > 90°, with a range from 67.88° to 109.3°. On the other hand, only 23% of PD participants had an ellipse angle < 90°, with a range from 75.08° to 165.81° (chi-square *p* = 0.03).

#### 3.2.2. Reaction Times

We evaluated reaction time for correct and incorrect trials in controls (Correct: 2.71 ± 1.01 s; Incorrect: 4.04 ± 1.51 s) and PD participants (Correct: 2.77 ± 0.98 s; Incorrect: 4.13 ± 1.3 s). No significant differences in reaction time were observed across groups (F = 0.173, *p* = 0.667, R^2^_m_ = 0.236); however, within groups showed increased reaction time for incorrect than correct trials in both controls (F = 854.881, *p* < 0.001) and PD groups (F = 1354.532, *p* < 0.001).

### 3.3. Binocular Vision

**Figure 5:**
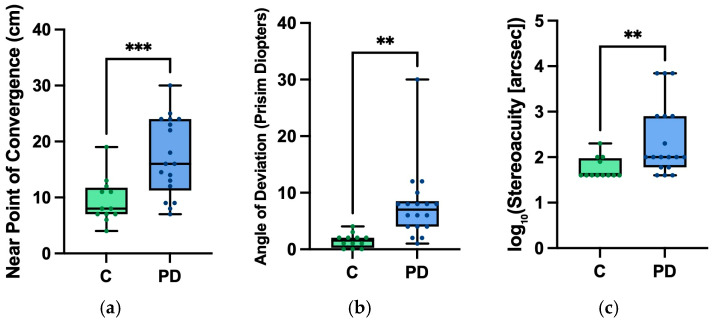
Plots of mean and standard deviation of control participants (“C”) vs. PD participants (“PD”) for (**a**) near point of convergence (cm) measured by RAF, (**b**) strabismus angle (prism diopters) at near, and (**c**) stereoacuity (arcsec). Statistical significance was established using the independent sample *t*-test (** = *p* < 0.01 and *** = *p* < 0.005).

We evaluated vergence, the strabismus angle at near, and stereopsis in controls versus PD participants. There was an increase in the NPC in our PD participants (17.1± 6.1 cm) compared to control participants (9.1 ± 4.0 cm) (t = 3.489, *p* = <0.001, d = 1.300) (Figure 5). In the alternate cover test, PD participants had a greater mean angle of deviation (7.7 ± 6.4 prism diopters) compared to control participants (1.5 ± 1.2 prism diopters) (t = 3.300, *p* = 0.001, d = 1.230) (Figure 5). Stereoacuity was worse in PD participants (2.3 ± 0.79 log arcsec) compared to controls (1.7 ± 0.24 log arcsec) (t = 2.672, *p* = 0.006, d = 0.996) (Figure 5).

### 3.4. Associations with Disease Severity

There was no significant correlation established between UPDRS scores and CCT global metrics: ellipse area (r = 0.108, *p* = 0.680, 95% CI [−0.406, 0.570]), long-axis length (r = 0.015, *p* = 0.953, 95% CI [−0.481, 0.504]), or short-axis length (r = 0.250, *p* = 0.333, 95% CI [−0.277, 0.661]).

No correlation was observed between vergence test results and UPDRS scores (r = −0.090, *p* = 0.732, 95% CI [−0.545, 0.406]). Additionally, there was no observed correlation between stereopsis and UPDRS scores (r = 0.003, *p* = 0.992, 95% CI [−0.476, 0.481]), or strabismic angle of deviation (r = 0.268, *p* = 0.282, 95% CI [−0.241, 0.662]).

## 4. Discussion

In this study, we evaluated both color vision using a computerized color vision assessment and examined binocular vision impairments in PD. Both color and binocular vision deficits are present in PD, with binocular vision impairments appearing more pronounced in our study population. Binocular vision testing showed convergence insufficiency type exotropia with demonstrated impaired stereopsis. Analysis of UPDRS scores showed no correlation between motor deficit severity and visual deficits. CCT testing demonstrated that our PD participants have larger ellipse areas, longer short axes, and ellipse angles more likely to exceed 90°. Comparisons of reaction window times between PD participants and control participants showed no significant differences for either correct or incorrect responses, indicating that differences in CCT metrics are not due to a delay in perception between PD and control groups. Specific deficits along individual discrimination vectors were not seen when comparing participant groups. Ellipse angles in PD showed a higher range of values and were more likely to be over 90° than those of our control participants. Altered ellipse angles potentially indicate specific directions of color vision loss, but specific conclusions are difficult to make with the wide range of values seen in the PD group. PD participants showed a larger ellipse area, indicating poorer color discrimination overall compared to our control participants; this finding is supported by prior studies utilizing these CCT in the PD population, which also found increased ellipse areas [29]. Notably, our control participants had a larger axial ratio (2.47 ± 0.76) than our PD participants (1.99 ± 0.91), in contrast to previous studies that reported a significant increase in the ellipse’s long-axis length with increased axial ratios [29], likely a result of significantly longer short-axis lengths (*p* = 0.009) in combination with a less-significant lengthening of the long axis (*p* = 0.034) in PD participants. This change in short-axis length and lower axial ratio can potentially be interpreted as more generalizable color-discrimination deficits in our PD participants, showing weaker color discrimination across a majority of vectors (creating a more circular ellipse), rather than deficits along specific confusion lines with stronger sensitivity to other vectors (creating a more oblong ellipse). This finding is supported by larger ellipse areas in PD participants, with no noted significant changes in individual vector lengths between groups, suggesting that the deficit in the PD group was more generalized. However, given the present changes in ellipse metrics are limited to area and short-axis length, this interpretation should be considered with caution; more extensive work is required to make definitive statements regarding the reason for the observed change in axis length.

Overall, the CCT showed non-specific changes in color vision in PD, with more general increases in ellipse area, ellipse short-axis length, and altered ellipse angle. Patterns of color vision disruption in PD have broadly been attributed to both parvocellular and cortical dysfunction. The parvocellular pathway is one of the major pathways through the lateral geniculate nucleus from the retina, functioning to transmit information on color and object form to the primary visual cortex [36]. Parvocellular dysfunction is suggested to result from retinal structural changes and reduced retinal dopamine [4]. PD patients also demonstrated significantly reduced dopaminergic amacrine cells in the retina [37]. Changes in retinal structures have been well described in PD patients, with OCT studies showing evidence of the retinal nerve fiber layer and ganglion cell layer thinning [38,39]; in one study, these changes were associated with color vision deficits and overall vision dysfunction in PD [40]. Retinal thinning has also been associated with disease severity and duration [6]. Broader cortical dysfunction involving posterior brain structures has also been described in late stages of PD [41,42], with decreased activation of the primary visual cortex during fMRI paradigms [42], which could lead to disruptions of higher-order visual processing, impairing color and contrast discrimination and potentially worsening effects of hypothesized parvocellular dysfunction.

Impairments in binocular vision were also assessed. PD participants showed significantly increased exotropic eye deviation at near and remote near point of convergence, consistent with convergence insufficiency. Decreases in stereoacuity were also significant in our PD participants. Vergence deficits in PD are well described in the literature [7,12,13,20]; central to this process is dopamine depletion, causing disinhibition and subsequent hyperactivity of the subthalamic nucleus, causing indirect inhibition of the supra oculomotor area of the midbrain [10,43]. Stereoscopic ability is a more complex process, functionally reliant on ocular alignment, but additionally reliant on the cerebral extrastriatal cortex [18,44]. PD patients with impaired stereopsis show extrastriate cortical atrophy [44], and studies have demonstrated correlations between stereopsis impairment and decreased cognitive function [45], suggesting mixed mechanisms of impairment in PD.

Exploratory outcomes of interest were associations between color vision and binocular vision changes with disease severity. As visual symptoms have been established in the prodromal stages of PD [8], visual function has emerged as a potential diagnostic marker in PD. The applicability of both more objective measures and more accessible testing is particularly desirable for PD, which is presently reliant on presentation with motor symptoms for clinical diagnosis. Markers of disease prior to significant irreversible neurodegeneration are especially meaningful, allowing for prompt initiation of treatment and lifestyle changes to slow progression, and longitudinal monitoring of disease progression and potential complications. Using UPDRS scores as a representation of disease severity, we found no significant correlation between disease severity and metrics of color vision deficits, consistent with previous findings [29]. No significant association was observed between binocular vision dysfunction and UPDRS scores, though increases in strabismic angle of deviation approached significance. These support that visual changes are part of a more complex pattern of cortical and subcortical dysfunction, rather than findings directly related to the progression of neurodegeneration.

Despite this study’s strengths, several limitations must be acknowledged. We did not calculate a priori sample size estimation prior to this study; this, with our modest sample size, may have limited ability to detect small statistical differences in testing performance or UPDRS correlation. One-sided tests were used for statistical analysis based on our a priori directional hypothesis; these findings should still be interpreted conservatively. Multiple comparisons were also used throughout the analysis, corrections were applied as indicated, but potential errors should be acknowledged. Our PD group included a mix of DBS and medication treatment. While all participants were tested in their dopaminergic “ON” state, the heterogeneous treatment strategies may impact group comparisons. In our examination of correlations with UPDRS scores, it should be acknowledged that UPDRS motor scores primarily reflect gross motor severity and may not accurately reflect retinal or binocular vision circuit impairment, potentially limiting the strength of the observed correlations.

Using the CCT in conjunction with clinical testing, we have demonstrated binocular vision dysfunction and nonspecific visuosensory changes in PD. PD participants show non-specific patterns of color vision deficiency, with increased ellipse area and increased short-axis length; broad color vision abnormalities, even when suboptimal motor skill is controlled for, support that CV deficits in PD are at least partially driven by earlier visual pathways. Future studies can explore potential directional patterns of deficits in PD using ellipse angles to clarify these findings. Binocular vision changes are present in PD, more commonly understood to be effects of efferent mechanisms. Associations between PD severity and changes in binocular and color vision are not generalizable, as both may be potentially present in earlier stages of PD. This variability indicates that while a pathologic connection is present, sensorimotor changes in PD are multifactorial, and understanding the precise pathophysiology and progression of visual dysfunction in PD requires more extensive testing.

## 5. Conclusions

PD participants exhibit binocular vision dysfunction with selective changes in color vision. Color vision changes in PD include increased ellipse area, increased axial length, and increased angular orientation; these changes are more variable in PD, likely due to mixed parvocellular and cortical dysfunction. Binocular vision dysfunction appears to be more common in PD, including both convergence insufficiency type exotropia and impaired stereopsis. Binocular vision changes are likely due to combined cortical and subcortical neurodegeneration. Both binocular and color vision dysfunction present independently of motor severity, suggesting multifactorial mechanisms of visual impairment. Findings emphasize the need for further investigation of visual dysfunction in PD and for routine visual testing in this population, regardless of symptom progression.

## Data Availability

The data presented in this study are available on request from the corresponding author due to privacy reasons. The requesting party will have to follow institutional agreement guidelines.

## Abbreviations

The following abbreviations are used in this manuscript:

PD	Parkinson’s Disease
CCT	Cambridge Color Test
CI	Convergence Insufficiency
UPDRS	Unified Parkinson’s Disease Rating Scale
NPC	Near Point of Convergence
FMT	Farnsworth–Munsell 100 Hue Test (FMT).
RAF	Royal Air Force Ruler
